# The Performance of Different Parametric Ultrasounds in Prostate Cancer Diagnosis: Correlation with Radical Prostatectomy Specimens

**DOI:** 10.3390/cancers16081502

**Published:** 2024-04-15

**Authors:** Adel Jawli, Ghulam Nabi, Zhihong Huang

**Affiliations:** 1 Division of Imaging Sciences and Technology, School of Medicine, Ninewells Hospital, University of Dundee, Dundee DD1 9SY, UK; 2 Department of Clinical Radiology, Sheikh Jaber Al-Ahmad Al-Sabah Hospital, Ministry of Health, Kuwait City 13001, Kuwait; 3 School of Science and Engineering, University of Dundee, Dundee DD1 4HN, UK

**Keywords:** prostate cancer, multiparametric ultrasound, shear-wave elastography, contrast-enhanced ultrasound

## Abstract

**Simple Summary:**

A systematic review assessed multiparametric ultrasound [mpUS] modalities in diagnosing prostate cancer via radical prostatectomy specimens. Between 2012 and 2023, eleven studies evaluated grayscale TRUS, SWE, CEUS, and mpUS. Sensitivity ranged from 37.7% to 55% for grayscale TRUS, 55% to 88.1% for SWE, 59% to 81% for CEUS, and 74% for mpUS, with specificities ranging accordingly. Notably, sensitivity for clinically significant prostate cancer was 55%, 73%, 70%, and 74%, respectively, with varying specificities. Quality Assessment of Diagnostic Accuracy Studies-2 [QUADAS-2] was used to evaluate bias and applicability. The review underscores the significance of mpUS modalities in prostate cancer diagnosis, highlighting their varying sensitivity and specificity in detecting both overall and clinically significant prostate cancer lesions.

**Abstract:**

Background: Prostate cancer is a prevalent cancer among men. Multiparametric ultrasound [mpUS] is a diagnostic instrument that uses various types of ultrasounds to diagnose it. This systematic review aims to evaluate the performance of different parametric ultrasounds in diagnosing prostate cancer by associating with radical prostatectomy specimens. Methodology: A review was performed on various ultrasound parameters using five databases. Systematic review tools were utilized to eliminate duplicates and identify relevant results. Reviewers used the Quality Assessment of Diagnostic Accuracy Results [QUADAS-2] to evaluate the bias and applicability of the study outcomes. Result: Between 2012 and 2023, eleven studies were conducted to evaluate the performance of the different ultrasound parametric procedures in detecting prostate cancer using grayscale TRUS, SWE, CEUS, and mpUS. The high sensitivity of these procedures was found at 55%, 88.6%, 81%, and 74%, respectively. The specificity of these procedures was found to be 93.4%, 97%, 88%, and 59%, respectively. This high sensitivity and specificity may be associated with the large lesion size. The studies revealed that the sensitivity of these procedures in diagnosing clinically significant prostate cancer was 55%, 73%, 70%, and 74%, respectively, while the specificity was 61%, 78.2%, 62%, and 59%, respectively. Conclusions: The mpUS procedure provides high sensitivity and specificity in PCa detection, especially for clinically significant prostate cancer.

## 1. Introduction

One of the most frequently diagnosed forms of cancer in males is prostate cancer [PCa], and it is the second leading cause of death after lung cancer [1]. Three approaches are available for exploring it: screening, histopathology, and medical imaging. The screening method involves the measurement of prostate-specific antigen [PSA] and the digital rectal exam [DRE]. PSA accuracy can be impacted by high PSA levels. In fact, when PSA levels are elevated, the test’s accuracy in detecting prostate cancer can decrease significantly. Therefore, a high PSA level should not be taken as a definitive diagnosis, and additional testing may be required to confirm any potential results [2]. The subjective nature of DRE examination leads to low sensitivity and specificity [3]. The standard method for detecting prostate cancer that has been used over the years is a biopsy, which is typically performed using transrectal ultrasound [TRUS]. However, biopsies have some drawbacks, such as a high likelihood of missing cancer and causing bleeding in the colon. In addition, more than half of patients who undergo systematic biopsies later require radical prostatectomy [RP] [4,5,6,7,8]. As a result, alternative methods are being employed to overcome these restrictions and enhance the precision of diagnosing prostate cancer. Magnetic resonance imaging [MRI] and ultrasound [US] are more efficient techniques for detecting prostate cancer. Despite being more sensitive than the US, MRI has its limitations. It is considerably more expensive and not suitable for all patients, including those with pacemaker ferromagnetic metals and those suffering from claustrophobia. Nevertheless, the US is a more cost-effective, non-invasive, and real-time procedure than MRI, which is suitable for all patients. To enhance the performance of PCa detection, various parametric methods have been developed in addition to grayscale ultrasound [GUS], such as Doppler ultrasound [DUS], elastography ultrasound [EUS], contrast-enhanced ultrasound [CEUS], and micro-ultrasound [9]. Grayscale ultrasound imaging, based on the density of organs, shows variations in brightness and darkness. Typically, prostate cancer presents isoechogenicity, meaning it has the same echogenicity as the surrounding tissue due to the prevalence of stromal fibrosis in prostate cancer tissue. However, if stromal fibrosis is minimal, hypoechoic PCa may be observed [10,11].

Doppler ultrasound, in the case of PCa, identifies abnormalities by examining the microvascular appearance of suspected lesions. These microvascular abnormalities are caused by increasing angiogenesis in neoplastic tissues [12,13,14,15,16]. The resolution of microvessels is typically lower than that of the Doppler shift, and this leads to reduced Doppler. Therefore, to visualize the inflow and outflow speed of blood vessels in suspected lesions, an intravenous micro-bubbles contrast agent is administered [17]. In the CUES, multiple measurements to differentiate between normal and abnormal tissues are determined. The Time-Intensity Curve [TIC] demonstrates the contrast agent signal over time after injection, displaying the Time to Peak [TTP] and Area Under the Curve [AUC]. Additionally, Rise Time [RT] and Mean Transit Time [MTT] are quantitative measurements that can indicate the presence of neoplastic lesions [18]. Although this test is effective, it may be limited in detecting a small lesion, and some patients may be allergic to the microbubble contrast agent [19,20].

Elastography is an important procedure used in ultrasound to measure tissue elasticity. It can be used to identify prostate cancer lesions, which are stiffer than surrounding tissue. In ultrasound, elastography has two different procedures: strain elastography [SE] and shear-wave elastography ultrasound [SWEUS]. SE provides a color map to differentiate between hard and soft tissue by inducing stress caused by transducer compression. While in SWE, an acoustic radiation force impulse [ARFI] is generated toward the tissues and assesses its stiffness based on the shear velocity [21].

This systematic review will study the performance of these modalities in prostate cancer detection. Two different studies completed a systematic review of ultrasound multiple parametric performance in detecting prostate cancer [22,23]. Postema et al. [22] examined how ultrasound can be utilized to detect prostate cancer and how these methods can be combined to improve detection accuracy, known as multiparametric ultrasound [mpUS]. They included elastography ultrasound, contrast-enhanced ultrasound [CEUS], and/or Doppler US in their research term besides the grayscale TRUS. Only one research database was used in their study, and the reference standards were biopsy and radical prostatectomy. The Alghamdi et al. [23] study was recently published; they analyzed the accuracy of various ultrasound parameters in PCa detection. They included CEUS, micro-ultrasound, and both elastography techniques associated with biopsy and radical prostatectomy as the reference standard.

This study aimed to systematically review the performance of the multiparametric ultrasound in prostate cancer detection. It will focus on radical prostatectomy specimens as a reference standard and shear-wave elastography ultrasound [SWEUS] instead of strain elastography [SE]. The objective of this study is to provide an accurate and comprehensive understanding of the performance of different parametric ultrasounds in the diagnosis of prostate cancer and its limitations.

## 2. Materials and Methods

A systematic review was conducted on various ultrasound parameters using five databases: PubMed, Scopus, Cochrane, MEDLINE, and Embase. The search results were filtered to include all articles published between January 2012 and the most recent articles available in 2023. This time frame was selected because the initial clinical study on SWEUS for prostate cancer detection was carried out in 2012 [24]. The primary focus of the research was to assess the effectiveness of various ultrasound techniques, such as grayscale ultrasound, shear-wave elastography ultrasound, Doppler ultrasound, contrast-enhanced ultrasound, and multiparametric ultrasound in detecting prostate cancer [PCa]. This study was registered with PROSPERO for the systematic review [PROSPERO Registration ID 467274]. The term MeSH was applied to all databases during the research in order to collect more articles related to the detection of prostate cancer using medical imaging modalities. Additionally, the search results were obtained based on the title and abstract of the articles. The search term included the following: “prostatic neoplasms/diagnosis” [MeSH Terms] OR “prostatic neoplasms/diagnostic imaging” [MeSH Terms] AND [“prostate cancer detect*” [Title/Abstract] OR “prostate cancer locali*” [Title/Abstract] OR “prostate cancer diagnos*” [Title/Abstract] AND [“ultrasound” [Title/Abstract] OR “ultrasonography” [Title/Abstract] OR “TRUS”[Title/Abstract] OR “transrectal ultrasound” [Title/Abstract] OR “gray scale ultrasound” [Title/Abstract] OR “doppler*” [Title/Abstract] OR “shear wave elastogra*” [Title/Abstract] OR “SWEUS” [Title/Abstract] OR “USWE” [Title/Abstract]. It should be noted that the search term included all possible keywords related to ultrasound techniques, and the use of NOT was avoided to ensure that no relevant articles were missed. This systematic review study followed the PRISMA guidelines and used the checklist in Tables S1 and S2 to ensure compliance.

Systematic review tools were employed to remove duplications and screen for eligibility criteria. The inclusion and exclusion criteria are mentioned in Table 1. The primary requirement for a study to be included is that it should involve a US parametric examination for patients enrolled for radical prostatectomy as a reference standard. The most important outcomes considered are sensitivity and specificity, while other outcomes like positive predictive value [PPV], negative predictive value [NPV], area under the ROC curve [AUC], and accuracy were also mentioned. No restrictions are based on the study’s country, race, or clinical institution.

The viewers used the Quality Assessment of Diagnostic Accuracy Result [QUADAS-2] tools to evaluate the bias and applicability of the study results. The tool consists of four sections: patient selection, index test, reference standard, and flow and timing [25]. Review Manager [RevMan] version 5.4 was used to achieve the quality assessment. The questions of the QUADAS-2 were explained by the authors and then answered accordingly [26,27].

## 3. Results

**Figure 1:**
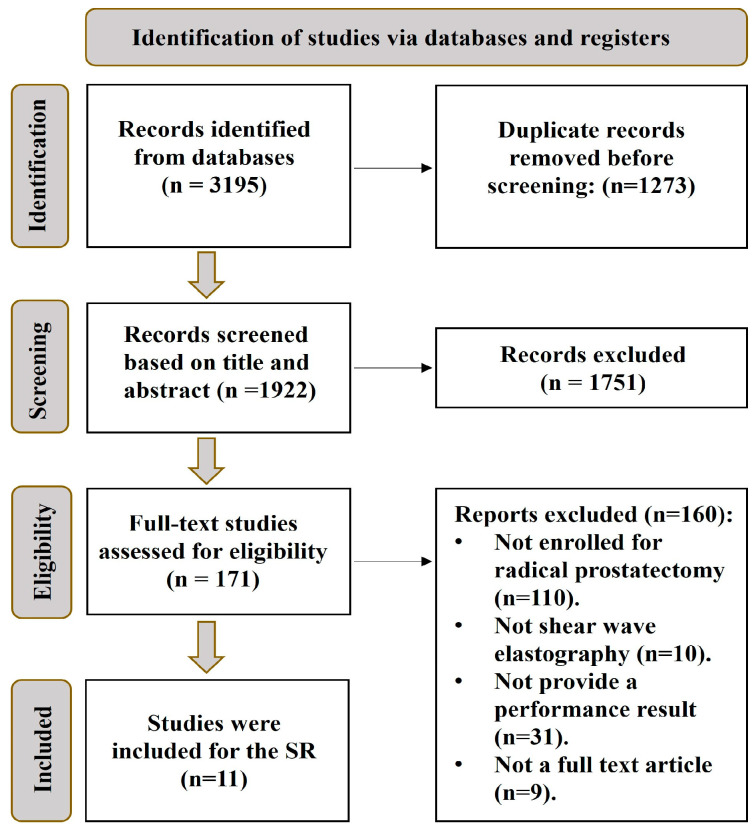
Prisma flow diagram.

The database results and eligibility studies used to identify the included studies in this research are briefly presented in Figure 1. Of the numerous studies screened, only 11 met the inclusion criteria and were included in this analysis. These studies were published between 2012 and 2023. Most of the excluded studies were related to MRI, CT, and PET scans for prostate cancer. A significant number of studies were excluded due to the absence of histopathological data from radical prostatectomy.

Among the studies included, SWEUS was the most used modality associated with the results of radical prostatectomy. Only one study compared the performance of mpUS in PCa detection with radical prostatectomy results. Unfortunately, no studies were found that assessed the performance of Doppler ultrasound in PCa detection with histopathological results from radical prostatectomy. The author’s name, year of publication, number of patients, lesion size, and study outcomes, such as sensitivity, specificity, PPV, NPV, accuracy, and AUC, of the included studies are presented in Table 2. The outcomes of each study were averaged, and the details were carefully considered.

### 3.1. Grayscale Ultrasound

The grayscale images in the provided studies have low sensitivity, likely due to the several imaging factors employed, such as the ultrasound frequency. Two studies [28,29] used low-frequency ultrasound for varying objectives. For instance, Zhu et al. [28] aimed to compare the accuracy of real-time elastography [RTE] with grayscale imaging. They used a bi-plane ultrasound probe to examine 56 patients and found that RTE has a slightly higher accuracy than grayscale ultrasound. Although the true-negative ratio is high in grayscale imaging, the true-positive ratio in RTE is higher.

The performance of two ultrasound frequencies, 5 MHz and 21 MHz, was compared in 25 patients [29]. High frequency provides higher image resolution, but the wavelength is short and unsuitable for high-depth organs. Ultrasound with lower frequency demonstrates low sensitivity, predictive value [PPV], and negative predictive value [NPV]. Nonetheless, in comparison with higher frequency ultrasound, both show close specificity. Not all patients started the ultrasound examination with the same probe to avoid bias in the result. Some started with the high frequency, and others started with the lower frequency.

Mannaerts et al. [30] examined the performance of grayscale mpUS for different stages of clinically significant prostate cancer [csPCa] based on the Likert score. A score of ≥3 referred to intermediate csPCa, while a score of ≥4 referred to high csPCa. Grayscale imaging is the primary technique used to localize prostate cancer, but its performance is low. Grayscale imaging was examined and compared with the RP result in the peripheral zone [PZ] and transition zone [TZ]. Grayscale imaging is less sensitive to localizing a high-grade csPCa in all prostate zones than intermediate csPCa grade. The data suggests that the detection of prostate cancer [PCa] in the peripheral zone [PZ] is significantly higher compared to the transitional zone [TZ], with a sensitivity of 57.1% and 21%, respectively. However, it should be noted that the specificity of detecting PCa in the PZ is comparatively lower at 62% compared to the TZ, which has a specificity of 83.2%.

### 3.2. Shear-Wave Elastography Ultrasound

Several factors need to be considered in shear-wave elastography performance, such as the cutoff value, which is the threshold to decide the malignancy, lesion location, and prostate size. Morris et al. (2021) [35] and Tyloch et al. (2023) [21] have a closed number of populations [36 and 30, respectively] using a similar ultrasound transducer, while only the patient position differed. The cutoff value in [21] was 35 kPa, and the sensitivity and specificity were 71.8% and 70.2%, respectively. In contrast, the cutoff value in [35] was 91.4 kPa, which increased the sensitivity, specificity, PPV, NPV, and AUC by 81%, 82%, 69%, 89%, and 0.48, respectively. Moreover, in [35], a 3D shear-wave elastography procedure was developed by acquiring more than 100 images with 1 to 1.5 angular spacing. Patients were scanned by SWEUS, ARFI, and B-mode. They showed that the mean elasticity of the peripheral zone [PZ] was lower than that in the central prostate by 69.1 kPa and 84.3 kPa, respectively. Moreover, the mean PCa lesion was 108 kPa.

Dai et al. [34] provided several performance outcomes based on several cutoff values, and the population was slightly higher. The sensitivity, specificity, and AUC were 81.3%, 82.4%, and 0.816, respectively, with a cutoff point of 84 kPa. The AUC was 0.776 for a cutoff point of 71 kPa, and the sensitivity and specificity were 78.1% and 76.5%, respectively. In the cutoff point of 60 kPa, the sensitivity and specificity were reduced to 68.8% and 70.6%, respectively. In addition, they show a correlation between SWEUS elasticity and the Gleason Group. The highest-grade group of PCa had an elasticity range of 84.1–117.2 kPa, while the lowest-grade group of PCa had an elasticity range of 41.6–67.3 kPa.

In 2023, Tyloch et al. [21] conducted a study comparing the performance of strain and shear-wave elastography, and they found that the Gleason score [GS] had a more significant impact on the sensitivity of results than the lesion size. For GS scores of 3, 4, and 5, the sensitivity was 54.6%, 81.36%, and 93.8%, respectively. The study showed that increasing the cutoff value could lead to high specificity but low sensitivity. Additionally, the average elasticity of benign, low-grade, intermediate-grade, and high-grade prostate cancer [PCa] was 36.43, 43.41, 55.93, and 66.81 kPa, respectively. Yet, it is essential to note that this was an average of both shear-wave and strain elastography.

Rouviere et al. [32] explored the impact of the size and location of prostate lesions on the results of elasticity testing. They found that 75% of patients did not have any elasticity data in the transition zone [TZ] due to a blind zone caused by an enlarged prostate gland. SWEUS detected more lesions in the peripheral zone [PZ] than in the TZ. This was particularly evident for lesions measuring 5 cm^3^ or larger. Although the stiffer lesions were found in the transitional zone [TZ] rather than in the peripheral zone [PZ], the elasticity data for the TZ was low. Finally, the study showed a difference in elasticity between the axial and sagittal planes, possibly due to increased pressure on the prostate gland during an axial scan.

**Figure 2:**
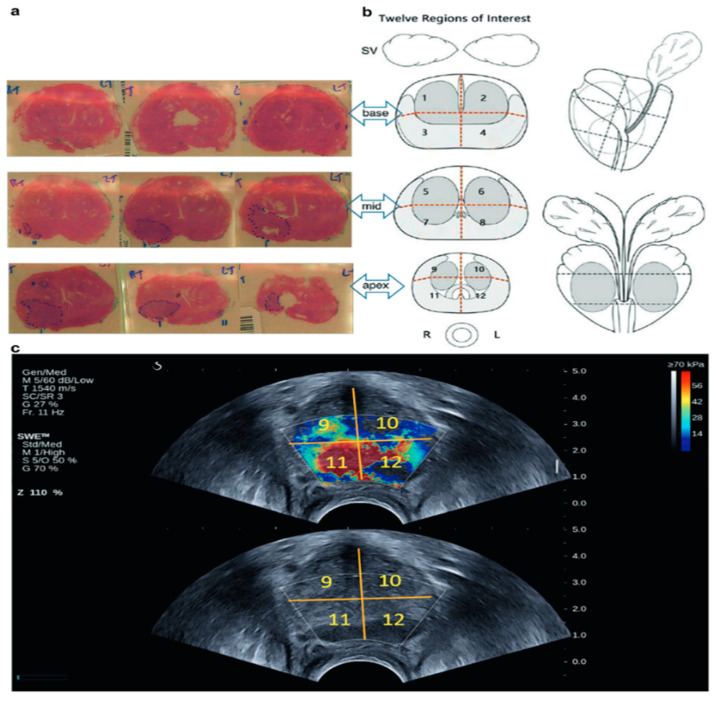
Histopathology results compared with 12-region SWE images obtained in a 73-year-old patient. (**a**) The whole set of prostate slices at 3 locations, including gland base, mid, and apexes. (**b**) A 12-region prostate imaging template. (**c**) Representative ultrasound images of the apex include the SWE image at the top and the b-mode image below [33].

Wei et al. [33] performed a study to evaluate the effectiveness of shear-wave ultrasound elastography in detecting significant prostate cancer. They measured the elasticity of the prostate gland based on the Gleason Score [GS], using a high cutoff value of 82.6 kPa. The results showed that the test’s sensitivity increased with higher GS and larger PCa lesion size. However, there was no significant difference in PCa elasticity according to PCa size. The study concluded that the elasticity of low, intermediate, and high-grade PCa was 91.6, 102.3, and 131.8 kPa, respectively, and the elasticity of each GS was also mentioned in the study. Figure 2 shows the true positive result of SWEUS in PCa detection correlated with RP result.

In a study by Mannaerts et al. [30], the effectiveness of SWEUS in detecting csPCa was examined. The study revealed that SWEUS may have limited sensitivity in detecting high csPCa grade across all prostate zones compared to intermediate grade. However, when examined by zones, SWEUS demonstrated higher sensitivity in detecting PCa in the PZ by 44.1% compared to the TZ by 37%, where specificity was equally observed in both zones.

### 3.3. Contrast-Enhanced Ultrasound

Two studies were carried out to assess the effectiveness of contrast-enhanced ultrasound [CEUS] in patients with prostate cancer [PCa] and to evaluate the results based on radical prostatectomy [36,37]. In both studies, a 2.4 mL bolus of SonoVue^®^ microbubble contrast agent was injected intravenously. In the first study [36], they recorded the contrast agent’s wash-on and wash-out for 3 min in the prostate gland. The study evaluated the performance based on three readings: the slope of the time-intensity curve [TIC], rise time [RT], and mean transit time [MTT]. The performance of these three readings was assessed, and the sensitivity, specificity, positive predictive value [PPV], and negative predictive value [NPV] were determined. The TIC reading provided a sensitivity of 82%, specificity of 96%, PPV of 100%, and NPV of 57%. The MTT reading provided a sensitivity of 73%, specificity of 78%, PPV of 89%, and NPV of 41%. Finally, the RT reading showed a sensitivity of 58%, specificity of 81%, PPV of 80%, and NPV of 37%. Notably, the number of cases analyzed in these three measurements was unequal. The study analyzed a total of 34 PCa lesions, with 30/34, 28/34, and 23/34 analyzed by TIC, MTT, and RT, respectively.

In the second study (Postema et al., 2020) [37], they compared the outcomes of CEUS and contrast-enhanced ultrasound Doppler imaging [CUDI] for clinically significant prostate cancer [csPCa]. A low-frequency TRUS probe was used, and a low contrast-specific power modulation pulse scheme by 3.5 MHz was applied with a mechanical index of 0.06 for contrast signal reading. The recording time was 2 min after contrast injection, and the inflow and outflow contrast readings were evaluated to diagnose csPCa. The sensitivity and specificity of CEUS, CUDI, and the combination were almost the same, with a slightly higher specificity of CEUS. Finally, it was found that increasing the cutoff value of the cancer detection rate leads to a decrease in sensitivity and an increase in specificity for both CEUS, CUDI, and both combined.

Mannaerts et al. [30] used CUES in the mpUS procedure. The ultrasound contrast agent was administered three times to define and detect the lesions, but no further details were provided on the type of reading used for lesion evaluation. The study detailed the outcome of CEUS of csPCa in the entire prostate gland, PZ, and TZ as intermediate and high grade. The sensitivity and specificity of CEUS in detecting PCa in the PZ were found to be 58.3% and 70%, respectively, while the sensitivity and specificity in the TZ were 37.65% and 79%, respectively.

### 3.4. Multiparametric Ultrasound

The increase in the average sensitivity of mpUS in comparison with ultrasound parametric individually was evident. Though only one [30] study provided the mpUS performance, it provides the result based on a high Likert score [≥4 and ≥3] and location of lesion [PZ and TZ]. Overall, the sensitivity of mpUS is higher than that of the other procedures individually. The specificity is low compared to other individual procedures. It has been noticed that the sensitivity of mpUS is higher in the detection PCa in PZ by an average of 73.5%, while in TZ, it is low by an average of 48.3. The average specificity of mpUS in PZ was 65.8%, and in TZ was 77.16%.

### 3.5. Other Results

Five research studies have been completed that correlate the ultrasound technique’s result with clinically significant prostate cancer. Three [21,33,34] were shear-wave elastography studies; one [37] used contrast-enhanced ultrasound, and the last used mpUS [30]. Table 3 shows the average outcome of these techniques in scanning a csPCa.

**Figure 3:**
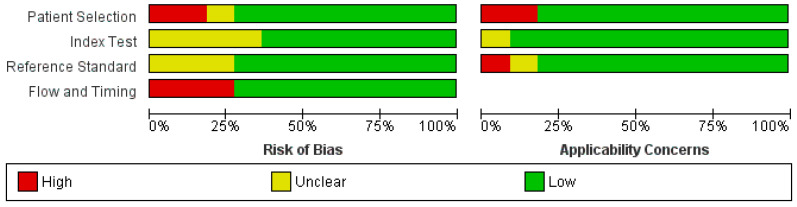
Risk of bias and applicability regarding graphs: review authors’ evaluation of domains presented as percentages across included studies.

**Figure 4:**
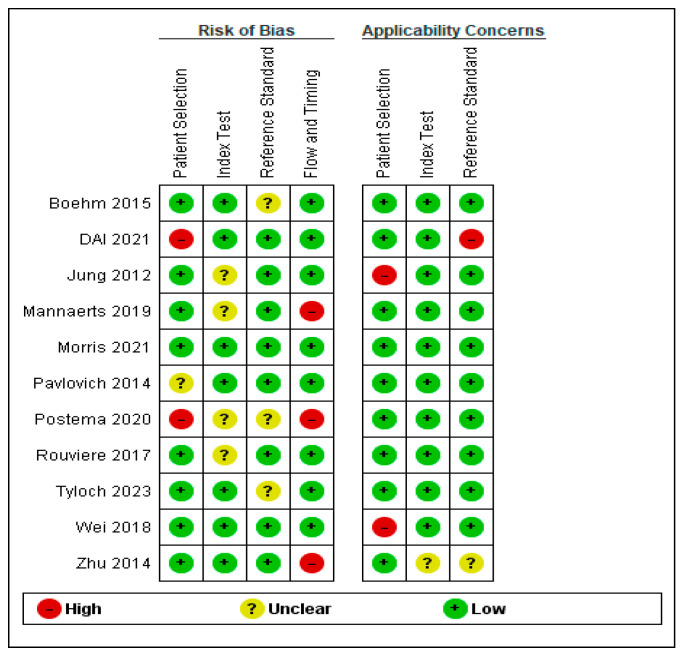
Risk of bias and applicability concerns summary: Review authors made judgments about each domain for each included study [21,28,29,30,31,32,33,34,35,36,37].

The quality assessment of study selection has shown a low risk of bias overall, as depicted in Figure 3 and Figure 4. However, two of the selected studies had a high risk of bias in patient selection as the selection was non-randomized [34,37]. Additionally, three studies provided a high risk of bias in flow and timing selection [28,30,37].

## 4. Discussion

The systematic review that is achieved is aimed at evaluating the diagnostic accuracy and performance of various ultrasound techniques, including grayscale, shear-wave elastography, and contrast-enhanced imaging, in detecting prostate cancer. The analysis involved assessing the sensitivity and specificity of these modalities based on several factors.

There is a debate about whether grayscale ultrasound is the best way to detect prostate cancer. While it is considered the gold standard in the US parametric for detecting neoplastic growths in the prostate, its ability to detect prostate cancer is limited after a biopsy has confirmed the presence of cancer, regardless of the number of samples taken [38]. Based on the studies included in this systematic review, the sensitivity of prostate cancer detection ranges from 25% to 56%, while the specificity ranges from 37.7% to 55%. These ranges may be increased due to the presence of significant prostate cancer [39]. High-frequency ultrasound is a valuable tool in detecting prostate cancer due to its exceptional sensitivity. Micro-ultrasound technology is a prime example of this, utilizing ultrasound waves with frequencies exceeding 29 MHz to achieve unparalleled resolution and tissue characterization when compared to traditional ultrasound methods [40,41]. In addition, the location and size of the PCa lesion have an influence on the sensitivity [28]. Studies have shown that a high percentage of PCa are hypoechoic, while others are isoechoic or hyperechoic [10,11,42,43,44], where this diversity in PCa echogenicity is due to the fibrosis of stromal cells, as was mentioned before. However, one study indicated that hypoechoic lesions have less stromal fibrosis compared to isoechoic PCa [10]. Another study [43] explained that hyperechoic PCa are rare and usually are ductal adenocarcinomas that contain central necrosis and calcification. The radiographic features of PCa under the US are well documented, but it is important to note that PCa’s appearance is mainly hypoechoic, which is one of the limitations in detection. Due to other prostatic issues that can also cause hypoechogenicity, such as prostatitis and benign prostatic hyperplasia, further testing may be required [45]. Thus, it is essential to identify the number of non-cancerous hypoechoic lesions for improved sensitivity while missing the number of neoplastic isoechoic lesions decreases sensitivity. This finding corresponds with previous studies that have examined the grayscale realization correlation with biopsy [46,47]. Conversely, Sauvain et al. [48] compared the efficacy of grayscale and power Doppler sonography in detecting prostate cancer. The results obtained were superior to those of the reviewed studies. However, the results were based on biopsy, and the specifics of the grayscale ultrasound procedure were not clearly outlined.

SWE has been used to assess the stiffness of lesions as an alternative regardless of the lesion echogenicity [24,49]. According to the current study, the sensitivity and specificity of SWEUS for detecting PCa generally range from 55 to 88.6% and 69.1 to 97.3%, respectively. The sensitivity and specificity of SWEUS for detecting csPCa range from 55 to 88.6% and 61 to 97.3%, respectively. As mentioned before, elasticity cutoff leads to either improvement or decrease in the performance of the SWEUS [21,34,35]; this is if the cutoff values of stiffness are lowered, it can identify more patients with the disease, but it can also lead to diagnosing more normal cases as abnormal, which is inaccurate [50]. Therefore, an optimal cutoff value must be determined to eliminate false-positive values. The lowest sensitivity may be due to missing SWE readings in PZ due to ultrasound heterogeneity. The highest specificity of SWEUS reading in TZ, as is shown in [30,32], is assembly due to the high stiffness of the TZ, which makes identifying the suspicious area effortless.

This systematic review discussed the shear-wave elasticity of benign, malignant, and csPCa. Therefore, this should be divided into general PCa and csPCa studies. The range stiffness of benign, PCa, and csPCa was between 11 and 65 kPa, 12 and 108 kPa, and 43.41 and 131 kPa, respectively, and this agreed with several studies [49,50,51,52]. The lowest range of prostate cancer was mentioned as caused by the depth range of the SWE [32].

Enhanced ultrasound is another advanced modality for prostate cancer detection that shows improving lesions in real-time, regardless of their echogenicity [53,54]. The sensitivity and specificity of the CUES procedure in detecting PCa ranged from 59% to 81% and 63% to 88%, respectively. The defining result in both studies [36,37] was high sensitivity, while [36] showed high specificity, probably from the increasing scan time after injection. It was agreed by [37] that the time of PCa detection in the interstate gland was insufficient. In addition, this high sensitivity is due to the increase in lesion size. A large lesion means more blood supply, which increases the blood flow. In this case, this enhances the perfusion pattern and increases the ability to visualize the lesion under CEUS.

We believe this study explained the performance of different ultrasound modalities in PCa in detail and associated it with the most accurate histopathological result. However, it is important to note that the study has a limitation that should be considered. Firstly, it should be pointed out that the number of selected studies was relatively small. As a result, the performance of the ultrasound parametric in the results showed a close correlation. As a second point, no studies were available that assessed the performance of Doppler ultrasound in detecting prostate cancer. This is because there is a lack of studies that correlate Doppler ultrasound with radical prostatectomy results. From 2012 to 2023, four studies evaluated the performance of Doppler ultrasound in detecting prostate cancer, but the outcomes were based on biopsy results only [55,56,57,58]. The review was also supposed to discuss the results of multiparametric ultrasound in detecting prostate cancer, but only one study included in the review provided outcomes based on radical prostatectomy results. According to the research results, multiparametric ultrasound studies in detecting prostate cancer were founded in 2013. Two studies [59,60] evaluated the achievements of multiparametric ultrasound based on biopsy results. The limited number of studies and data, especially with multiparametric ultrasound, may make it difficult to compare different procedures. Another limitation of the studies included is that the biopsy confirmed the diagnosis of prostate cancer, which could cause bias.

Based on the results of this systematic review, we recommend that future studies should aim to assess the performance of multiparametric ultrasound in detecting prostate cancer with radical prostatectomy results. Furthermore, there is a need for further studies on grayscale, contrast-enhanced ultrasound, and Doppler ultrasound in detecting prostate cancer and their performance based on radical prostatectomy results. What is more, it has been noticed that no study, to our knowledge, addressed the influence of echogenicity on the shear-wave. There were only a few studies that focused on clinically significant prostate cancer in all modalities, so further studies are requested to detect clinically significant prostate cancer. In addition, the accuracy of prostate detection was not provided in 8 out of 11 studies, which should be considered in future studies.

## 5. Conclusions

This systematic review presents qualitative results from studies examining multiple ultrasound modalities for prostate cancer detection based on histopathological results from radical prostatectomy. The modalities assessed include grayscale imaging, shear-wave elastography, and contrast-enhanced ultrasound. When used in combination, these techniques enhance the performance of ultrasound parameters in detecting prostate cancer, providing various quantitative information that significantly matches in vivo results.

## Figures and Tables

**Table 1:** Inclusion and exclusion criteria.

Criteria	Inclusion	Exclusion
Settings	All Countries	None
Participants	Male patients [all ages] with a suspicion of prostate cancer, based on biopsy	No radical prostatectomy enrolled
Modality	Ultrasound parametric such as: Grayscale, shear-wave elastography, Doppler, contrast-enhanced ultrasound, and combination multiparametric ultrasound	Studies that did not examine these devices.
Outcomes	Studies that report sensitivity, specificity, positive predictive value, negative predictive value, accuracy, and AUC.	Studies that do not report at least sensitivity or specificity.
Study Type	In vivo studies Prospective and retrospective studies Randomized Clinical trial non-randomized	In vitro studiesReview articlesSystematic review
Publication Type	Journal articles	Conference abstract, study protocol, report, dissertation, books, and non-professional journal
Publication Year	Publication date 2012 and after	Publication date before 2012
Language	English	All other languages

**Table 2:** Number of studies in each modality, with the average outcomes in each study.

Modality	Author [Year]	Number of Patients	Lesion Size	Sensitivity %	Specificity %	PPV	NPV	Accuracy %	AUC %
Grayscale ultrasound	Zhu et al., 2014 [28]	56	≤5 mm, ≥5 mm	40.2	93.4			77.0	
	Pavlovich et al., 2014 [29]	25	≥5 mm	37.7	65.4	48.1	55.2		
	Mannaerts et al., 2019 [30]	50	≥5 mm	55	61	59	57		
Shear-wave elastography ultrasound	Boehm et al., 2015 [31]	60	≥5 mm	81.1	69.1			47.2	
	Rouvière et al., 2017 [32]	31	≤5 mm, ≥5 mm	61	67.1	77.8			76
	Wei et al., 2018 [33]	212	≥5 mm	88.6	97.3	86.3	97.8	96	97
	Mannaerts et al., 2019 [30]	50	≥5 mm	55	61	59	57		
	Dai et al., 2021 [34]	42	≥5 mm	76	77.2				75.7
	Morris et al., 2021 [35]	36		81	82	69	89		84
	Tyloch et al., 2023 [21]	30	≥5 mm	65.3	70.2				
Contrast enhanced ultrasound	Jung et al., 2012 [36]	20		71	88	89	45		
	Mannaerts et al., 2019 [30]	50	≥5 mm	59	63	62	60		
	Postema et al., 2020 [37]	133	≥5 mm	81	64				78
Multiparametric ultrasound	Mannaerts et al., 2019 [30]	50	≥5 mm	74	59	65	70		

**Table 3:** The performance of different ultrasound modalities in clinically significant prostate cancer diagnoses.

Modality	Sensitivity %	Specificity %
Grayscale ultrasound	55	61
Shear-wave elastography ultrasound	73	78
Contrast-enhanced ultrasound	70	62
Multiparametric ultrasound	74	59

## Data Availability

The data presented in this study are available within this article.

## Supplementary Materials

The following supporting information can be downloaded at: https://www.mdpi.com/article/10.3390/cancers16081502/s1, Table S1: PRISMA 2020 Main Checklist; Table S2: PRIMSA Abstract Checklist. Reference [61] is cited in the Supplementary Materials.
